# Introgression of *QTL hotspot* regions enhances grain yield and maize lethal necrosis resistance in elite maize lines

**DOI:** 10.1038/s41598-026-53717-8

**Published:** 2026-05-21

**Authors:** Veronica Ogugo, Vijay Chaikam, Michael S. Olsen, Yoseph Beyene, L. M. Suresh, Liezel Herselman, MacDonald Bright Jumbo, Prasanna M. Boddupalli, Manje Gowda

**Affiliations:** 1 International Maize and Wheat Improvement Center (CIMMYT), World Agroforestry Centre (ICRAF), United Nations Avenue, Gigiri, P.O. Box 1041-00621, Nairobi, Kenya; 2 Department of Plant Sciences, University of the Free State, P.O. Box 339, Bloemfontein, 9300 South Africa; 3 Crop Science Division, Bayer, 800 N. Lindbergh Blvd, St. Louis, MO 63167 USA; 4 International Crops Research Institute for the Semi-Arid Tropics (ICRISAT), BP 320, Bamako, Mali

**Keywords:** Maize, MLN resistance, Disease severity, Grain yield, MABI, QTL, Genetics, Molecular biology, Plant sciences

## Abstract

Maize lethal necrosis (MLN) poses a severe threat to maize production in eastern and southern Africa, causing significant yield losses. In this study, marker-assisted backcross introgression (MABI) was used to introgress major-effect MLN resistance Quantitative Trait Loci (QTL). These QTLs located on chromosomes 3 and 6, were introgressed into 14 MLN-susceptible CIMMYT maize lines. Ten Kompetitive Alelle Specific PCR (KASP) SNP markers closely linked to three validated *QTL-hotspot* regions were applied for foreground selection, with at least two hotspots polymorphic across all donor–recipient combinations. Foreground and background selection enabled fast tracking of MLN resistance alleles and recovery of near-recurrent parent genomes. The resulting BC₄F_3_ introgressed lines exhibited markedly reduced MLN severity under artificial inoculation, with several lines showing a 50% reduction relative to their recurrent parents. Testcrosses of these lines demonstrated yield advantages of 2–4 t/ha under MLN pressure compared with original parental lines, while maintaining comparable performance under optimum conditions. Notably, introgressed derivatives of CML312, CML539, and CZL052 displayed both enhanced MLN resistance and superior yield performance, with CZL052-derived testcrosses achieving nearly two-fold yield gains under severe MLN stress. Importantly, equivalence trials confirmed that MLN resistance was improved without compromising resistance to gray leaf spot, turcicum leaf blight, or common rust. These findings validate the effectiveness of QTL-based conversion for enhancing MLN resistance in elite breeding lines and demonstrate the potential of these improved lines as robust parental sources for developing MLN-resilient hybrids adapted to eastern and southern Africa.

**Supplementary Information:**

The online version contains supplementary material available at 10.1038/s41598-026-53717-8.

## Introduction

 Maize (*Zea mays* L.) is the world’s second most cultivated cereal and, with rice and wheat, supplies over 40% of global food calories^1,2^. Demand is expected to rise by 50% by 2050^3^, yet biotic and abiotic stresses increasingly constrain production. In Sub-Saharan Africa, maize covers over 44 million hectares and feeds more than 300 million people^4^. However, average yield was just 2.1 t/ha—less than half the global mean—due to drought, degraded soils, low inputs, and pests and diseases^1,5,6^. Viral diseases are particularly devastating, sometimes causing complete crop failure. Major maize viruses in Africa include *maize streak virus*,* maize chlorotic mottle virus* (MCMV), *sugarcane mosaic virus* (SCMV), *maize chlorotic dwarf virus*,* maize dwarf mosaic virus*, and *maize rough dwarf virus*^7–10^.

Among viral diseases in maize, Maize Lethal Necrosis (MLN) poses the greatest threat, capable of causing up to 100% yield loss in smallholder systems. MLN arises from a synergistic co-infection of MCMV and SCMV. First reported in Kenya in 2012^11^, the disease has since spread across Eastern Africa, imposing an estimated annual economic burden of >$339 million on smallholder farmers^12^. MCMV, a single-stranded RNA virus in the *Tombusviridae* family, is transmitted primarily by thrips and beetles, but also mechanically, with rare cases of seed and soil transmission^13,14^. Originally identified in Peru in 1973, MCMV has since been reported in the USA, Latin America, and China^15–17^. SCMV, a *Potyviridae* virus, is mainly aphid-transmitted but can also spread mechanically. While infections by either virus alone are usually mild, co-infection results in severe symptoms—leaf mottling, stunting, premature senescence, necrosis, sterility, and crop death before tasseling^11,18^.

Following the MLN outbreak, surveys by the International Maize and Wheat Improvement Centre (CIMMYT) and the Kenya Agriculture and Livestock Research Organization (KALRO) revealed that over 90% of commercial and pre-commercial hybrids in the region were highly susceptible to MLN^19,20^. This triggered urgent breeding efforts to identify resistance sources. These are now being used to introgress MLN resistance into susceptible but agronomically superior backgrounds^21,22^.

Genetic studies revealed that resistance to MLN is governed by a few major effects and several minor effect quantitative trait loci (QTL)^19,23–25^. A genome-wide association study (GWAS) identified single nucleotide polymorphisms (SNPs) explaining 8–10% of the phenotypic variance, with combined effects accounting for ~ 30%^19^. Subsequent biparental mapping validated these associations and revealed major QTL on chromosomes 3, 6, and 9, with effects ranging from 3.9% to 43.8% of phenotypic variation^19,26,27^. Additional studies confirmed major-effect loci on chromosomes 3 and 6, consistently detected across diverse genetic backgrounds^24,28^. These loci represent prime candidates for introgression of MLN resistance QTL into elite maize lines.

Marker-assisted backcrossing (MABC) provides a powerful tool to introgress major-effect QTLs. By exploiting molecular markers tightly linked to target QTL, MABC accelerates the transfer of resistance alleles into elite cultivars while minimizing linkage drag^29^. The approach enables early and cost-effective selection, reducing breeding cycles and improving precision. MABC has been successfully applied to enhance resistance against several maize diseases—for example, introgression of *qHSR1* for head smut^30^, *ZmCCT-H5* for stalk rot, flowering regulation, and yield stability^31,32^, and pyramiding of major genes for maize rough dwarf disease and gray leaf spot resistance into elite inbreds^33,34^.

In East Africa, many commercially successful hybrids derive from parent lines that are highly susceptible to MLN. These hybrids are difficult to replace quickly because they combine high yield potential, drought tolerance, and resistance to multiple foliar diseases. A practical breeding strategy is therefore to introgress MLN resistance QTL into these susceptible parents through MABC. This approach enhances MLN resistance while retaining the superior agronomic performance of popular hybrids, extending their utility and ensuring food security as new MLN-resistant germplasm is developed. MABC is a structured breeding approach that combines foreground, recombinant, and intensive background selection to introgress a target gene/QTL while rapidly recovering the recurrent parent genome (typically > 95% within a few backcross generations), often aiming to develop near-isogenic lines or EDVs. In contrast, marker-assisted introgression or marker-assisted backcross introgression (MAI/MABI) focuses primarily on transferring target genomic regions or haplotypes into a recipient background using markers, with limited background selection; as a result, larger donor segments are often retained and overall genome recovery is lower, making it more suitable for rapid trait deployment when fine mapping or stringent genome recovery is not the immediate priority. The current study is more of MABI approach where we tried to introgress 1–3 Mb genomic region from donor lines to recipient parent.

In this study, we applied MAI/MABI approach to introgress major-effect MLN resistance QTL into elite but MLN-susceptible CIMMYT maize lines. Using ten SNP markers tightly linked to resistance loci on chromosomes 3 and 6, we deployed five MLN-tolerant donors to transfer resistance into 14 recurrent elite lines. The specific objectives were to: (i) introgress major-effect MLN resistance QTL on chromosomes 3 and 6 into drought-tolerant but MLN-susceptible elite lines through MABI; (ii) Assess the level of MLN resistance in the improved lines relative to their recurrent parents, and (iii) Evaluate testcross hybrids derived from the introgressed lines for both agronomic performance and MLN resistance compared to the original parents. Together, these objectives aim to deliver MLN-resistant lines and hybrids without compromising yield or adaptive traits. This will help sustain the value of commercially successful germplasm and support long-term maize productivity in Eastern Africa.

## Materials and methods

### Germplasm used

Between 2013 and 2015, CIMMYT evaluated a large number of inbred lines and identified a subset with tolerance to MLN (https://www.cimmyt.org/news/update-cimmyt-maize-inbred-lines-and-pre-commercial-hybrids-with-potential-resistance-to-maize-lethal-necrosis-mln-2/). From these, five MLN-tolerant inbred lines (DTPYC9-F46-1–2-1–2-B, CLYN261, CML543, CML574 and CLRCY034) were selected as trait donors for population development and later in resistance introgression (Table 1). Among these, DTPYC9-F46-1–2-1–2-B and CLYN261 belong to heterotic groups A and the rest belon to heterotic group B. Eight recipient lines included CML312, CML444, CML507, CML539, CML540, CML544, DTPWC9-F67-1–2-1–2, LaPostaSeqC7-F64-2–4-1-1. Donor lines are known for wide adaptation to the region and tolerance to MLN, while recipient lines are known for high yield potential, strong combining ability, drought tolerance, low-soil nitrogen stress tolerance, and resistance to multiple foliar diseases. This combination provided an ideal foundation for introgressing MLN resistance into agronomically superior but MLN-susceptible elite lines, ensuring both disease resistance and retention of desirable agronomic traits in the breeding pipeline.

### Genotyping

Plants were tagged, and leaf samples were collected after three weeks of planting. From each tagged plant, four 6 mm leaf discs were collected. The samples were dried using silica gel. Ten KASP SNPs linked to three QTL for MLN tolerance were selected. These QTL had been consistently identified in earlier studies^19,20,23^. The selected markers were used to assess polymorphism between donor and recipient lines and to support foreground selection. Genotyping was done at LGC, UK (https://www.lgcgroup.com). The resulting marker data enabled the selection of plants homozygous for favorable alleles at the targeted MLN resistance QTLs (Supplementary Table S1). For the background selection, leaf discs from donor parent, recipient parent, and BC_4_F_3_ plants were sent to Diversity Arrays Technology (DArT), Australia, for genotyping using the Maize DArTag 3.3 K EiB (2.0) panel developed by the CGIAR Excellence in Breeding (EiB) platform (https://excellenceinbreeding.org/toolbox/services/mid-density-genotyping- service). This publicly available panel comprises 3,305 DArTag markers, derived from over 10,000 genetically diverse maize inbred lines.

**Table 1:** Donor and recipient maize inbred lines used in this study, their heterotic group classification, and SNPs linked to major-effect QTL for MLN resistance on chromosomes 3 and 6, including favorable and unfavorable alleles.

Donor line	HG	Haplotype/QTL	SNP marker	Favorable allele	Unfavorable allele	Recipient line
DTPYC9-F46-1–2-1–2-B	A	*MLN_03.133*	PZA02299_16	AA	GG	CML539, LaPostaSeqC7-F64-2–4-1-1
			PZA00363_7	GG	AA	
			S3_133048570	CC	TT	
CLYN261	A	*MLN_03.133*	PZA02299_16	AA	GG	CML312, CML540, CML544, DTPWC9-F67-1–2-1–2, LaPostaSeqC7-F64-2–4-1-1
			S3_133048570	CC	TT	
		*MLN_03.140*	S3_146250249	GG	TT	
			S3_146363360	TT	CC	
			S3_146602134	TT	CC	
CML543	B	*MLN_03.133*	PZA02299_16	AA	GG	CML202, CML489, CML546, CML574, CZL052, CLRCY034, CML444
			PZA00363_7	GG	AA	
			S3_133048570	CC	TT	
		*MLN_03.140*	S3_146250249	GG	TT	
			S3_146363360	TT	CC	
			S3_146602134	TT	CC	
		*MLN_06.20*	PZA03047_12	GG	AA	
			S6_21007530	GG	AA	
			S6_21008211	CC	TT	
CML574	B	*MLN_03.133*	PZA02299_16	AA	GG	CML444
			PZA00363_7	GG	AA	
			S3_133048570	CC	TT	
CLRCY034	B	*MLN_03.133*	PZA02299_16	AA	GG	CML507
			PZA00363_7	GG	AA	
			S3_133048570	CC	TT	
		*MLN_03.140*	S3_146250249	GG	TT	
			S3_146363360	TT	CC	
			S3_146602134	TT	CC	

*HG* heterotic group.

### Introgression of the QTL-hotspot genomic regions

The MLN resistance QTL-hotspot region was introgressed independently into elite recipient lines using a MABI approach. In heterotic group A, donor parent *CLYN261* was used to introgress MLN resistance into five elite recurrent lines (Table 1). Additionally, *DTPYC9-F46-1–2-1–2-B* served as the donor for *CML539* and *LaPostaSeqC7-F64-2–6-2-2*. In heterotic group B, *CML543* was used as the donor for seven elite recipient lines, while *CML574* and *CLRCY034* each contributed MLN resistance to one recipient line (Table 1).

In the backcrossing scheme, the recipient parent was consistently used as the female and the donor parent as the male across all generations. Crosses were conducted at CIMMYT’s Kiboko Research Station, Kenya, between 2014 and 2017. To accelerate population advancement, two planting cycles per year were implemented. F₁ hybrids were first generated by crossing donor and recipient parents, followed by backcrossing to the recurrent parent to produce BC₁F₁ progenies in 2014. Selection in the BC₁F₁ generation was based solely on agronomic traits (e.g., vigor, height, ear traits, standability, and overall phenotype). Three additional backcross generations (BC₂F₁, BC₃F₁, and BC₄F₁) were advanced during 2014–2015. Foreground selection was initiated at the BC₂F₁ generation using SNP markers tightly linked to major MLN resistance QTL. Haplotype *qMLN3_133* includes four SNPs with favorable alleles AA/GG/CC/AA (vs. GG/AA/TT/GG), *qMLN3_146* comprises three SNPs with TT/CC/TT (vs. GG/TT/CC), and *qMLN6_021* on chromosome 6 contains three SNPs with GG/GG/CC (vs. AA/AA/TT) across their respective markers (Supplementary Table S1). Genotyped plants carrying favorable alleles were tagged, and selected ears were advanced by planting ~ 21 seeds per row, from which 14–16 plants per row were genotyped.

In 2016–2017, BC₄F₁ progenies were selfed twice to generate BC₄F₃ families. Foreground selection was applied at both BC₄F₁, BC₄F_2_ and BC₄F₃ stages, while background selection using genome-wide SNP markers was conducted at the BC₄F₃ stage to identify plants with the highest recurrent parent genome (RPG) recovery and phenotypic similarity to their recurrent parents. The percentage of RPG recovery was calculated following the method described previously^35,36^. In simple terms, RPG was computed using the formula: G = [(X + 0.5Y) × 100]/N, where N is the total number of SNPs that were polymorphic between the parents, X is the number of SNPs that were homozygous for the recurrent parent allele, and Y is the number of SNPs that were heterozygous for the two parental alleles. From each cross, the two best introgressed lines were selected and advanced for evaluation of MLN resistance and agronomic equivalence relative to their recurrent parent.

### Evaluation of MLN introgressed lines and their testcrosses

To assess the efficacy of introgressed QTLs and the equivalence of introgressed lines to their recurrent parents, testcrosses were developed at CIMMYT’s Kiboko Research Station. Inbred tester CKDHL120312 (heterotic group B) was crossed with donor lines, recurrent parents, and their BC₄F₃ introgressed derivatives from heterotic group A (*CLYN261*,* DTPYC9-F46-1–2-1–2*,* CML312*,* CML539*,* CML540*,* CML544*,* DTPWC9-F67*, and *LaPostaSeqC7-F64*). Tester CKDHL120918 (heterotic group A) was used to cross lines from heterotic group B (*CML202*,* CML543*,* CML444*,* CML507*,* CML489*,* CML546*,* CML574*,* CZL052*, and *CLRCY034*), along with their introgressed BC₄F₃ versions. Both testers were fixed for all three MLN resistance QTL.

To evaluate the effect of introgressed QTL on MLN resistance, the introgressed lines and their testcross hybrids were evaluated in 2018 for their response to MLN under artificial infestation. The test cross hybrids were also evaluated under natural infestation at an MLN hotspot region. In all these trials, each experimental unit consisted of a two-row plot, 4 m in length, with 0.75 m between rows and 0.25 m between plants within a row. Two seeds were planted per hill and later thinned to one plant per hill at three weeks after emergence, targeting a final population of 53,333 plants per hectare. Fertilizer management included a basal application of di-ammonium phosphate (DAP) at planting, providing 60 kg N and 60 kg P₂O₅ per hectare. Six weeks after emergence, all plots were top-dressed with an additional 60 kg N per hectare. Weed control was maintained through a combination of manual weeding and herbicide application.

All phenotyping trials for MLN resistance under artificial infestation were conducted at CIMMYT’s MLN screening facility in Naivasha, Kenya. For the inbred line trial, a total of five donor lines, 14 recurrent parent lines, and 31 selected introgressed lines (the two best lines per MABI cross) were evaluated to assess disease severity and grain yield. Testcross hybrids generated using the 31 introgressed lines along with test cross hybrids for donor and recurrent parents, were evaluated under artificial MLN inoculation in 2017. Additionally, the same testcrosses were also evaluated for MLN resistance in the natural MLN hotspot region of Babati in Tanzania across two seasons, 2017 and 2018.

The hybrids were also evaluated under optimal conditions across multiple environments in 2018 to determine both the efficacy of the introgressed QTLs under MLN pressure and the agronomic equivalence of the introgressed lines to the recurrent parents in the absence of MLN. These environments included Kiboko, Kitale, and Thika in Kenya under optimum management conditions in 2018. All trials were conducted with an alpha-lattice experimental design with three replications per genotype.

### Data collection and statistical analysis

At each location, data were recorded on a per-plot basis for the following traits: days to 50% anthesis (AD; days from planting until 50% of plants shed pollen), days to 50% silking (SD; days from planting until 50% of plants produced silks), and anthesis–silking interval (ASI; calculated as SD – AD). Plant height (PH) was measured as the distance in centimeters from the base of the plant to the insertion of the first tassel branch. Ear height (EH) was measured as the distance from the base to the node bearing the uppermost ear; both PH and EH were recorded on 10 representative plants per plot. At harvest, grain moisture (MOI; determined using a moisture meter from grains sampled at the center of five representative ears per plot) was recorded. Grain yield (GY) was computed from the field weight using a shelling percentage of 80% and adjusted to 12.5% grain moisture. Analyses of variance (ANOVA) for individual environments (under both MLN pressure and optimum conditions) were conducted according to the restricted maximum likelihood procedure using the multi-environment trial analysis program in R (META-R)^37^ based on the following linear mixed:

$$\:{\boldsymbol{y}}_{\boldsymbol{j}\boldsymbol{k}\boldsymbol{l}}=\:\boldsymbol{\mu\:}+{\boldsymbol{R}\boldsymbol{e}\boldsymbol{p}}_{\boldsymbol{j}}+{\boldsymbol{B}\boldsymbol{l}\boldsymbol{o}\boldsymbol{c}\boldsymbol{k}}_{\boldsymbol{k}}\left({\boldsymbol{R}\boldsymbol{e}\boldsymbol{p}}_{\boldsymbol{j}}\right)+{\boldsymbol{G}\boldsymbol{e}\boldsymbol{n}}_{\boldsymbol{l}}+{\boldsymbol{\epsilon\:}}_{\boldsymbol{j}\boldsymbol{k}\boldsymbol{l}}$$

where $\:{\boldsymbol{y}}_{\boldsymbol{j}\boldsymbol{k}\boldsymbol{l}}$ is the trait of interest; $\:\boldsymbol{\mu\:}$ is the mean effect; $\:{\boldsymbol{R}\boldsymbol{e}\boldsymbol{p}}_{\boldsymbol{j}}$ is the effect of $\:{j}^{th}$ replicate, $\:{\boldsymbol{B}\boldsymbol{l}\boldsymbol{o}\boldsymbol{c}\boldsymbol{k}}_{\boldsymbol{k}}\left({\boldsymbol{R}\boldsymbol{e}\boldsymbol{p}}_{\boldsymbol{j}}\right)$ is the effect $\:{k}^{th}$ of the incomplete block within the $\:{j}^{th}$ replication, $\:{\boldsymbol{G}\boldsymbol{e}\boldsymbol{n}}_{\boldsymbol{l}}$ is the effects of the $\:{l}^{th}$ genotype, and $\:{\boldsymbol{\epsilon\:}}_{\boldsymbol{j}\boldsymbol{k}\boldsymbol{l}}$ is the error associated with the $\:{i}^{th}$ replicate, $\:{k}^{th}$ of the incomplete block, and $\:{l}^{th}$ genotype, which is assumed to be normally and independently distributed, with mean zero and homoscedastic variance. In this model, genotypes were considered fixed effects, calculating best linear unbiased estimator (BLUE), whereas replications, blocks within replications, were considered as random effects. To estimate variance components, all factors were considered random effects.

**Fig. 1:**
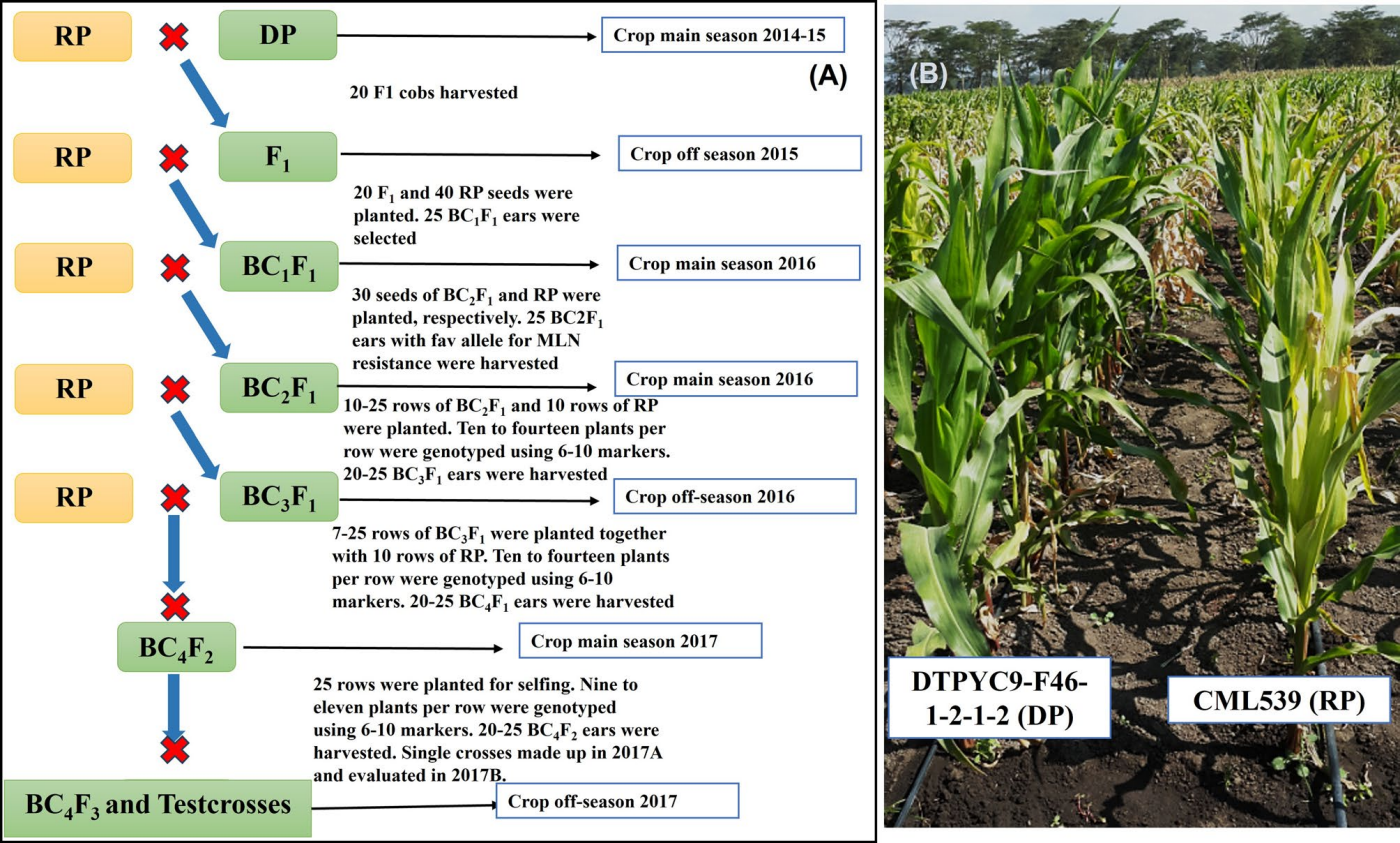
MABI scheme for introgression of the MLN resistance “QTL-hotspot” genomic regions. (**A**) Details of the MABI scheme adopted, (**B**) Expression of MLN disease severity in donor parent (DP) and recurrent parent (RP) lines.

**Table 2:** Estimation of phenotypic and genotypic variance components for MLN disease severity (MLNDS) and grain yield (GY) evaluated under MLN and optimum conditions in different locations.

Trait	σ^2^_*P*_	σ^2^_G_	σ^2^_e_	H^2^	LSD_0.05_	CV (%)
Naivasha - lines perse under MLN						
MLNDS	2.00	1.94**	0.06	0.97	1.04	2.05
Naivasha - Testcross hybrids under MLN						
MLNDS	0.87	0.78**	0.09	0.90	0.84	14.95
GY	3.87	3.24**	0.62	0.84	1.89	31.4
Babati1 - Testcross hybrids under MLN						
MLNDS	0.13	0.08*	0.05	0.59	0.48	28.72
GY	1.66	1.27**	0.40	0.76	1.76	26.09
Babati2 - Testcross hybrids under MLN						
MLNDS	0.12	0.11*	0.01	0.92	1.90	19.00
GY	0.69	0.22*	0.47	0.32	2.04	62.72
Optimum - Testcross hybrids						
Kiboko - GY	0.24	0.11**	0.14	0.44	1.03	17.80
Kitale - GY	2.30	1.47**	0.83	0.64	2.55	26.12
Thika - GY	0.24	0.05*	0.19	0.20	1.23	20.26

*σ*^2^_P_ phenotypic variance, *σ*^2^_G_ Genotypic variance, *σ*^2^_e_ error variance, *H2* heritability, *LSD*_0.05_ least significant difference at 5%, *CV* coefficient of variation, * and ** significant at *p* = 0.05 and *p* = 0.01 level, respectively.

## Results

### Markers for foreground and background selection

A total of seven recipient lines from heterotic group A and nine from heterotic group B were selected for the introgression of MLN resistance QTLs (Table 1). Selection in the BC₁F₁ generation was based exclusively on agronomic traits, including plant vigor, plant height, ear characteristics, standability, and overall phenotypic acceptability, to ensure recovery of the recurrent parent background. No selection for MLN resistance was conducted at this stage. The haplotype *qMLN3_133* comprises four SNPs with favorable allele combinations of AA/GG/CC/AA and corresponding unfavorable alleles of GG/AA/TT/GG for markers PZA02299_16, PZA00363_7, S3_133-48570, and PZD00015_5, respectively. The haplotype *qMLN3_146* consists of three SNPs with favorable alleles TT/CC/TT and unfavorable alleles GG/TT/CC for markers S3_146250249, S3_146363360, and S3_146602134, respectively. Similarly, the haplotype *qMLN6_021* on chromosome 6 includes three SNPs with favorable allele combinations GG/GG/CC and unfavorable alleles AA/AA/TT for markers PZa3047_12, S6_21007530, and S6_21008211, respectively (Supplementary Table S1). Foreground selection was carried out using a panel of these ten KASP markers. Only the markers that were polymorphic between the donor and recurrent parent pairs were utilized for selection (Table 1; Supplementary Table S1, Table S2A). Among the ten KASP markers tested, three SNPs—PZA02299_16, PZA00363_7, and S3_133048570—were polymorphic between donor line *DTPYC9-F46-1–2-1–2* and the recurrent parents *CML539* and *LaPostaSeqC7-F64-2–6-2-2*. In another set of crosses involving donor line *CLYN261* and recipient lines, five SNPs were identified as polymorphic (Table 1). For heterotic group B, nine SNPs were found polymorphic between donor line *CML543* and recurrent parents. Additionally, three SNPs were polymorphic between donor–recipient pairs *CML574* × *CML544* and *CLRCY034* × *CML507* (Table 1). Overall, at least two QTL-hotspot regions showed polymorphism in all donor–recipient combinations (Table 1; Supplementary Table S1). For example, in *CML202*,* CML489*, and *CML546*, SNPs linked to *qMLN3_133* and *qMLN6_021* were utilized for foreground selection. Markers linked to *qMLN3_133* (chromosome 3) were consistently polymorphic across all donor–recurrent parent combinations, whereas markers from the other two hotspot regions displayed polymorphism only in specific parental combinations.

For background selection, among the 3305 SNPs tested on the selected donor and recipient parents, 1800 markers were used after quality check, among them, 1160 (*CML312/CLYN261*), 942 (*CML540/CLYN261*), 1157 (*CML544/CLYN261; DTPWC9-F67/CLYN261; CML539/DTPYF-46*), 942 (*LPSC7-F64/CLYN261; LPSC7-F64/DTPYF-46*), 1119 (*CML202/CML543*), 1116 (*CML489/CML543*), 1141 (*CML546/CML543*), 1146 (*CML574/CML543*), 918 (*CZL052/CML543*), 1142 (*CLYCR034/CML543*), 1158 (*CML444/CML543*), and 1160 (*CML507/CLYCR034*) markers showed informative polymorphisms that were used for background selection (Supplementary Table S2B). For the background selection, the number of polymorphic markers distributed per chromosome varied from 43 to 177.

**Fig. 2:**
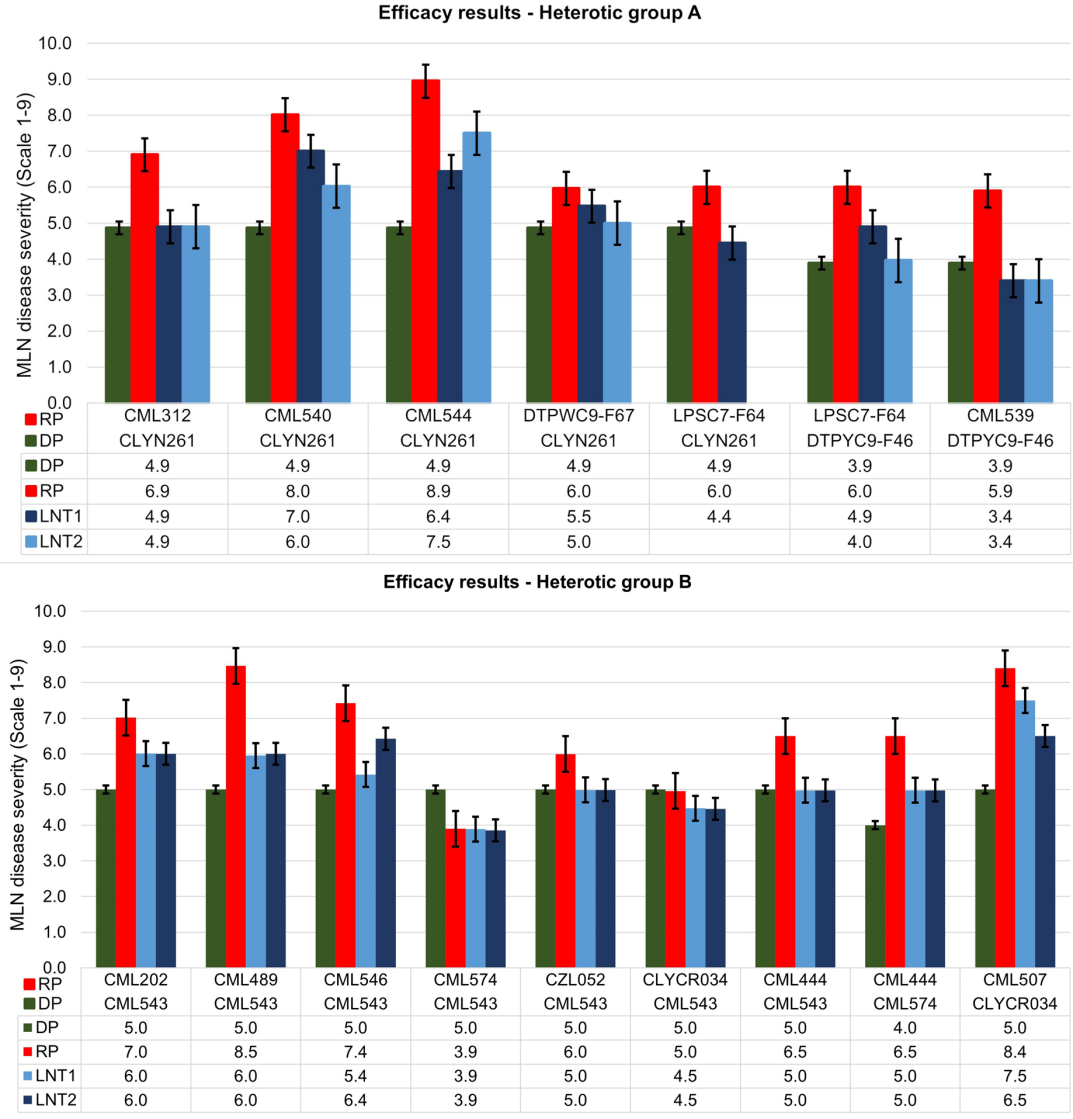
Efficacy results on per se performance - Evaluation of donor parent, recurrent parent, and their introgressed lines under artificially inoculated MLN conditions. LNT1 and LNT2 represent lethal necrosis tolerant line 1 and line 2.

### Phenotypic evaluation of introgression lines for their efficacy

With an objective to assess the efficacy of MABI for transferring the *QTL-hotspots*, 31 introgressed lines were evaluated for MLN disease severity (Fig. 2) along with their donor and recurrent parents. Evaluation of MLN resistance showed consistent differences between donor parents, recurrent parents, and the introgressed lines (LNT1 and LNT2) (Fig. 2). In heterotic group A, recurrent parents exhibited high MLN disease severity scores (6.0–8.9), whereas donor parents consistently had lower scores (3.9–4.9). The introgressed lines (LNT1 and LNT2) expressed reduced severity compared to their recurrent parents, with scores ranging between 3.4 and 7.5, indicating a clear improvement in MLN resistance. The highest reduction in MLN disease severity was observed for introgressed lines derived from *CML539* × *DTPYC9-F46* (5.9 vs. 3.4) and *CML312* × *CLYN261*(6.9 vs. 4.9). Similar trends were also observed in heterotic group B, where recurrent parents recorded high disease severity (7.0–8.5) relative to donor parents (5.0). The introgressed lines exhibited lower disease severity (3.3–6.5), approaching the levels of the donor parents, particularly in *CLYCR034* × *CML543*,* CML574 × CML543* and *CZL052 × CML543* combinations. Based on LSD values, introgressed lines from five crosses in heterotic group A and six crosses in group B showed significantly lower MLN disease severity than their original recurrent parents (Table 2; Fig. 2). “Introgressed lines outperformed selected elite lines under MLN disease pressure, indicating enhanced resistance conferred by the introgressed QTL (Figure S1) Overall, the introgression of MLN resistance QTLs confirms the effectiveness of the conversion process in improving MLN resistance.

### Phenotypic evaluation of testcrosses for their efficacy

**Fig. 3:**
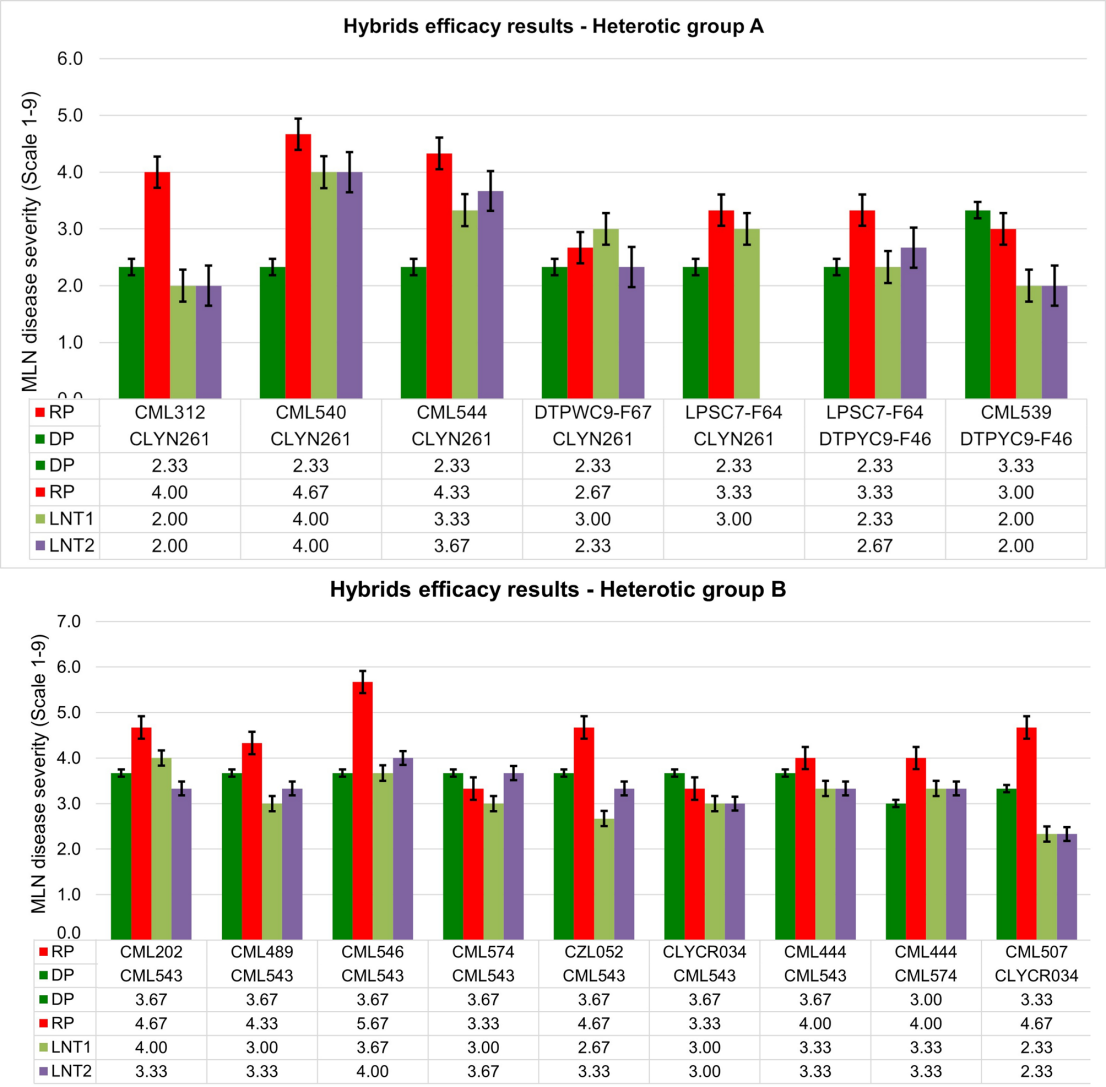
Efficacy results on testcross hybrids performance for MLN disease severity - Evaluation of testcrosses of donor parent, recurrent parent, and their introgressed lines under artificially inoculated MLN conditions. LNT1 and LNT2 represent lethal necrosis tolerant testcross hybrids 1 and 2.

**Fig. 4:**
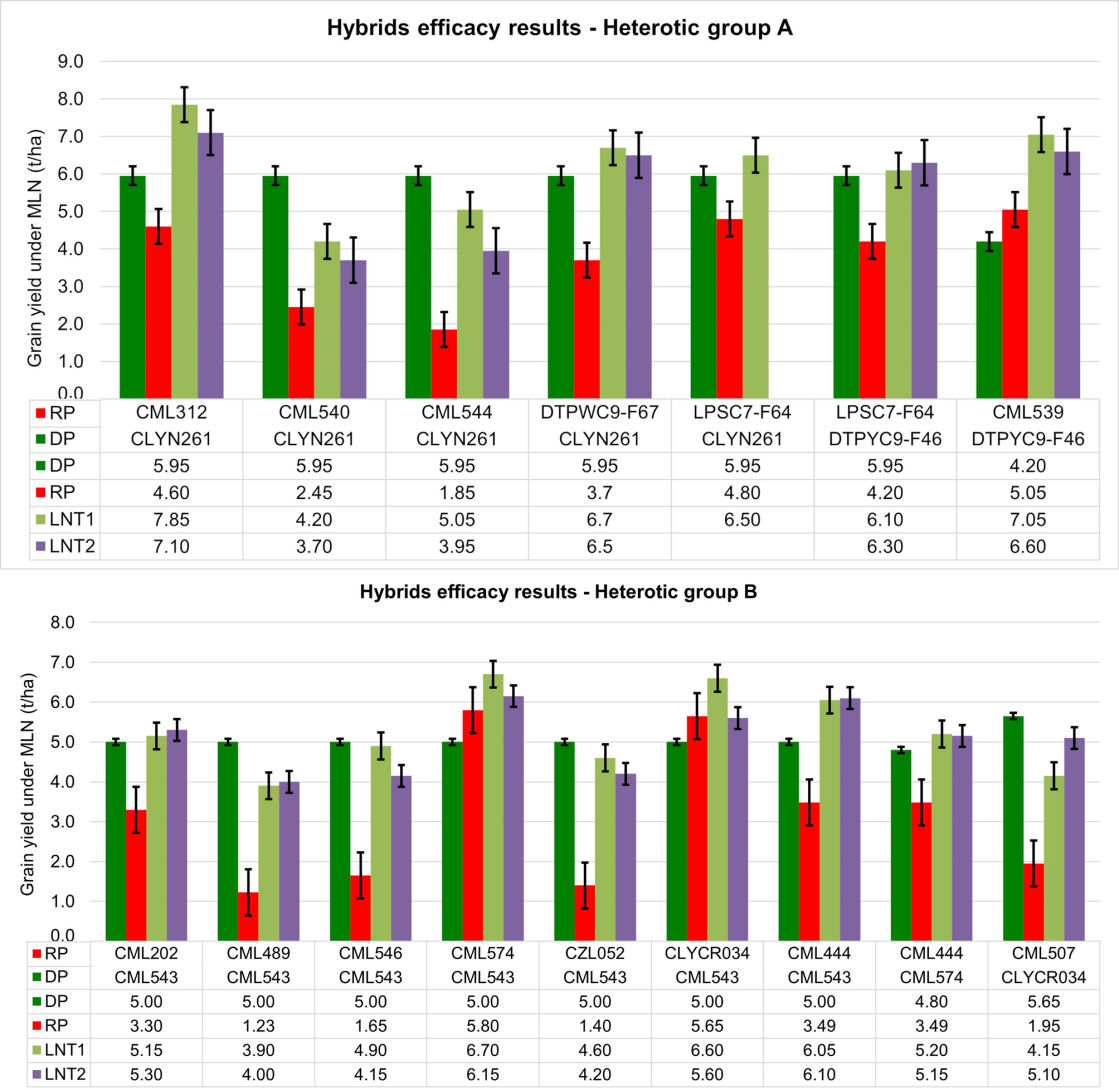
Efficacy results on testcross hybrids performance for grain yield under MLN disease pressure - Evaluation of testcrosses of donor parent, recurrent parent, and their introgressed lines under artificially inoculated MLN conditions for grain yield. LNT1 and LNT2 represent lethal necrosis tolerant testcross hybrids 1 and 2.

Evaluation of MLN resistance among testcross hybrids exhibited consistent differences between the testcrosses of donor parents, recurrent parents, and the introgressed lines (Fig. 3). In heterotic group A, recurrent parents exhibited moderate MLN disease severity scores, whereas donor parents consistently had lower scores (2.33–3.33). The testcrosses of introgressed lines demonstrated reduced severity scores compared to recurrent parents, with scores ranging between 2.0 and 4.0, indicating a clear improvement in MLN resistance. The highest reduction of MLN disease severity was observed for introgressed versions of CML312. The efficacy of transferring MLN resistance *QTLs* in a cross between *CLYN261* × *CML312* revealed a 50% reduction in MLN disease severity in introgressed lines compared to the recurrent parent CML312 (Fig. 3) and an increase in GY of 2.25 tons/ha under MLN disease pressure (Fig. 4). Overall, the testcross hybrids revealed a considerable reduction in MLN disease severity in introgressed lines compared to their original recurrent parents, except for *CLYN261* × *DTPWC9-F67* cross (Fig. 3).

Similar trends were observed in heterotic group B, where testcrosses of recurrent parents recorded moderately high severity compared to test crosses of donor parents (3.00–3.67.00.67). The testcrosses of introgressed lines exhibited lower disease severity (2.3–4.0). Higher levels of MLN score reduction were recorded in testcrosses of introgressed versions of CML507, CZL052, and CML489. Overall, the introgression of MLN resistance QTLs led to significant reductions in disease severity across both heterotic groups, confirming the effectiveness of the conversion process in improving MLN resistance. Based on the LSD value for MLN disease severity (0.85), introgressed lines from four crosses in heterotic group A and five in group B showed significantly lower MLN severity than their recurrent parents (Table 2; Fig. 3).

GY under MLN stress varied significantly among testcross hybrids of donor parents, recurrent parents, and introgressed lines (LNT1 and LNT2) (Fig. 4). In heterotic group A, testcrosses of recurrent parents consistently showed low yields under MLN (1.85–4.80 t/ha), while test crosses of donor parents recorded higher yields (4.20–5.95 t/ha). Testcrosses of introgressed lines (LNT1 and LNT2) showed substantial yield advantages over recurrent parents, with hybrids yielding between 3.70 and 7.85 t/ha. Particularly testcross hybrids from introgressed lines of CML312 (LNT1, 7.85 t/ha) and CML539 (LNT1, 7.05 t/ha) exhibited the highest yields, clearly outperforming both donor and recurrent parents. Testcross hybrids of introgressed lines of CML544 (5.05 vs. 1.85 t/ha), DTPWC9-F67 (6.7 vs. 3.7t/ha) showed the highest yield gains under MLN stress compared to testcross hybrids of their recurrent parents.

Similar trends were observed in heterotic group B, where recurrent parents yielded poorly (1.23–3.65 t/ha) compared to donor parents (4.80–5.65 t/ha) under MLN stress. The introgressed lines (LNT1 and LNT2) consistently outperformed recurrent parents, with yields ranging from 4.15 to 6.60 t/ha. Testcross hybrids of introgressed lines of CML574 (LNT2, 6.15 t/ha) and CML507 (LNT1, 6.60 t/ha) achieved the highest yields under MLN stress. Particularly, testcross hybrids of introgressed lines of CML543 (4.00 Vs 1.23 t/ha), CML546 (5 vs.1.65t/ha), CZL052 (4.60 vs1.40 t/ha) and CML507 (5.65 vs. 1.95 t/ha) achieved the highest yields under MLN stress compared to testcross hybrids of their recurrent parents, indicating effective recovery of productivity through the introgression of MLN resistance. Overall, the introgressed lines significantly enhanced hybrid performance under MLN pressure in both heterotic groups, approaching or surpassing the donor parent yields.

Analyses of variance on grain yield and other agronomic traits under MLN disease (Table 2, Supplementary Table S3, S4 and S5) and under optimum management (Table 2, Supplementary Table S6, S7 and S8) revealed significant variation and moderate to high heritability estimates for each location. Comparison of introgressed lines against their original recipient parents revealed no significant changes for agronomic traits like AD, SD, PH, EH, ER, husk cover and for foliar diseases like GLS, common rust and TLB under optimum management.

**Fig. 5:**
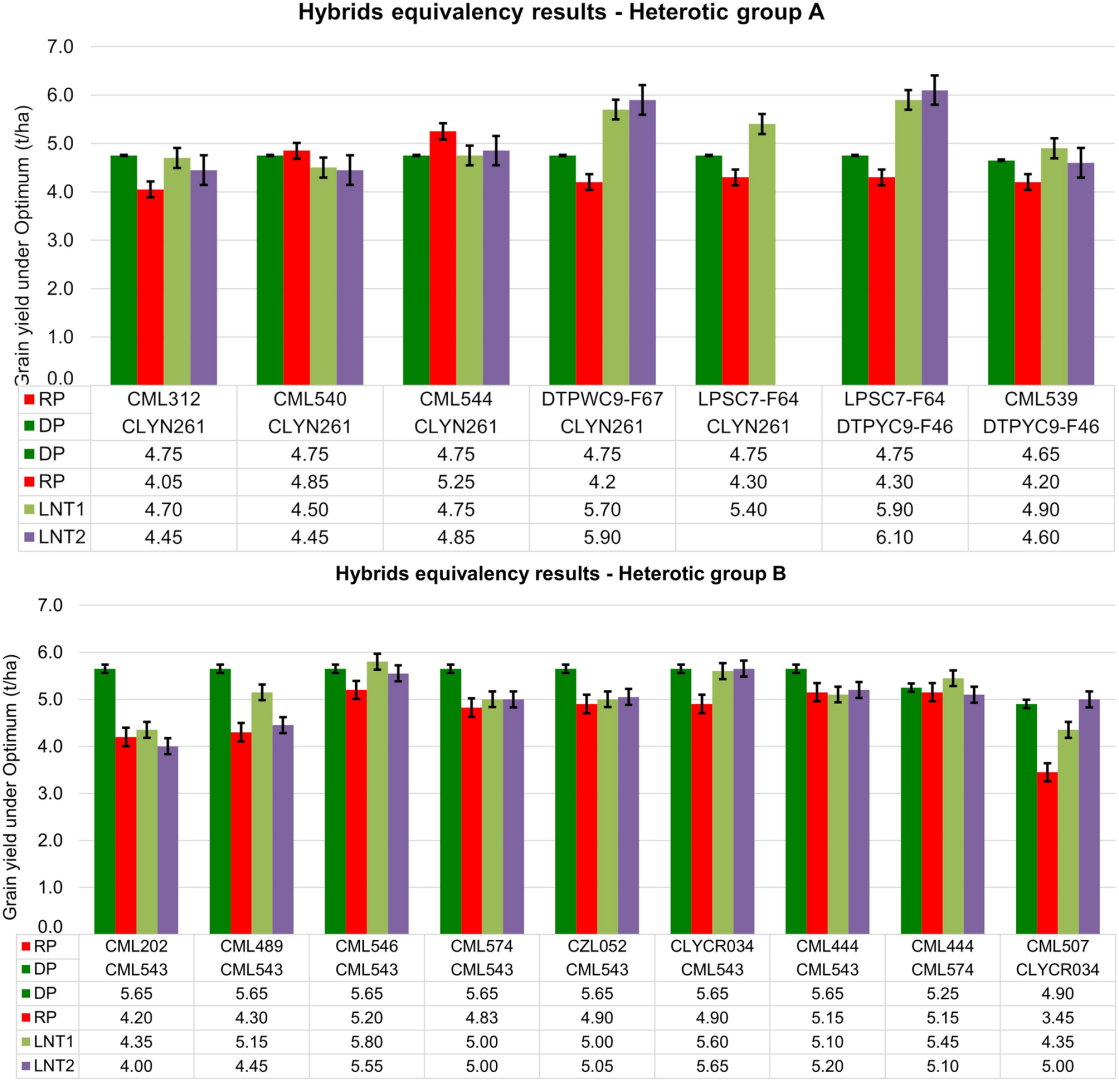
Equivalency test - Evaluation of testcross hybrids of donor parent, recurrent parent, and their introgressed lines under optimum conditions for grain yield. LNT1 and LNT2 represent lethal necrosis tolerant testcross hybrids 1 and 2.

GY equivalency in the absence of MLN disease pressure showed minimal differences among the testcrosses of donor parents, recurrent parents, and introgressed lines (LNT1 and LNT2) across both heterotic groups (Fig. 5). In heterotic group A, average yields of testcrosses of recurrent parents ranged between 4.05 and 5.25 t/ha, while donor parents yielded 4.65–4.75 t/ha. The testcrosses of introgressed lines performed comparably, with yields of 4.45–6.10 t/ha. LPSC7-F64 (LNT2, 6.10 t/ha) and DTPWC9-F67 (LNT2, 5.90 t/ha) and CML539 showed slightly higher yields than both donor and recurrent parents. For CML312 and CML540, the testcrosses of introgressed lines showed higher yield compared to the recurrent parent but less than the donor parent. For CML544, the yield of testcrosses of introgressed lines showed less yield than the recurrent parent but same/slightly higher yield than the donor parent.

Similarly, in heterotic group B, testcross hybrids of donor parents yielded 4.90 to 5.65 t/ha, while recurrent parents ranged between 4.20 and 5.20 t/ha. Testcross hybrids of introgressed lines matched these levels yielding 4.00–5.80 t/ha. Notably, testcross hybrids of introgressed lines of CML546 (LNT1, 5.80 t/ha) and CLYCR034 (LNT1, 5.65 t/ha) maintained yields equivalent to donor parents, while outperforming recurrent parents. For the rest of the lines, testcross hybrids even though did not perform better than the donor parents, the introgressed lines did slightly better or similar to the recurrent parent. These results demonstrate that the introgression of MLN resistance QTLs into elite lines did not negatively affect hybrid yield potential under optimum conditions.

## Discussion

Climate change has intensified biotic a^11^ nd abiotic stresses across sub-Saharan Africa, making the development of maize hybrids with multiple stress tolerance a breeding priority^20^. MLN disease, first reported in Kenya in 2011^11^, has caused devastating yield losses, restricted seed movement due to transmission through seed and insect vectors. This also exposed the vulnerability of many stress-tolerant and agronomically superior maize hybrids. To address this challenge, conventional breeding alone is inadequate, and the integration of modern tools such as genomics, high-throughput phenotyping, and systems modeling is essential^38,39^. MABI offers a strategic, quick response by introgressing MLN resistance alleles into elite, high-yielding hybrids, enabling rapid genetic gain while preserving key agronomic traits. The resulting lines provide a resilient foundation for hybrid development, ensuring food security and strengthening the maize supply chain in eastern and southern Africa.

Marker assisted backcrossing (MABC) or MABI is particularly effective for improving disease resistance, as phenotypic selection for resistance can be unreliable due to viral mutations and strong year-to-year environmental variation. Unlike phenotypic approaches, molecular markers are environment-independent, allowing precise and consistent introgression of resistance loci into elite germplasm^40–42^. MLN resistance is largely quantitative, controlled by a few major-effect and several minor-effect QTL^19,20,23,26–28^, making it especially amenable to MABC. The present study used seven SNPs on chromosome 3 (*PZA02299_16*,* PZA00363_7*,* S3_133048570*,* PZD00015_5*,* S3_146250249*,* S3_146363360*, and *S3_146602134*) and three SNPs on chromosome 6 (*PZA03047_12*,* S6_21007530*, and *S6_21008211*), all located within *QTL-hotspot* regions linked to MLN disease resistance in maize^19,20,23,27^. The strong alignment between phenotypic resistance and the presence of favorable alleles at these loci underscores their value in breeding pipelines. Indeed, 31 BC₄F_3_ introgressed lines carrying these favorable alleles exhibited MLN resistance, providing direct evidence for the functional relevance of these hotspots. Similar successes have been documented in other cereals, such as the introgression of *Pi-b* and *Pi-kh* blast resistance genes into rice via MABC^43^. Nevertheless, the expression of QTL can be affected by epistatic interactions or linkage drag in new genetic backgrounds^44,45^. Therefore, the development of tightly linked or functional markers for MLN resistance remains a priority to enhance selection accuracy and reduce the number of individuals required in each backcross generation.

The consistent polymorphism observed for SNP markers linked to *qMLN3_133* across all donor–recipient parent combinations highlights the robustness of this *QTL hotspot* and its potential utility as a stable source of resistance. This finding is in line with earlier studies reporting chromosome 3 regions as critical for MLN resistance^20,23,26,27^. The partial polymorphism observed for the other two QTL hotspots suggests that their utility may depend on the genetic background of the parents, further emphasizing the importance of multiple resistance loci to broaden the genetic base of MLN resistance.

Resistance QTL can sometimes carry negative effects on yield or plant growth^46,47^, and maintaining heterosis after line conversion requires a high level of genetic background recovery. In our study, although introgressed chromosomal regions were relatively large, no yield penalties were observed in the introgressed lines. This is probably because of MLN resistance genes residing within the target QTL regions^23,48,49^. The RPG recovery in the introgressed lines ranged from 60% in CML489 to 98% in CML540, with other genetic backgrounds showing recovery rates between 83% and 90% (Supplementary Table S2B). These results are consistent with findings in other crops, where, for example, 91.6% RPG recovery was achieved in rice (*Oryza sativa* L.) while pyramiding blast resistance genes into elite Basmati^50^, and > 90% recovery was reported in chickpea after just three backcross generations^51,52^. The use of a relatively large number of SNPs (788 to 1160) ensured that undesired donor genome segments were minimized, a critical step to avoid linkage drag and maintain the agronomic performance of elite germplasm. Similar marker-assisted breeding strategies have proven effective in other maize breeding programs targeting traits such as drought tolerance and disease resistance^53,54^.

Phenotypic evaluations of introgressed lines provided strong evidence of improved MLN resistance. Across both heterotic groups, recurrent parents were highly susceptible, whereas donor parents showed moderate resistance. Introgressed lines consistently demonstrated reduced disease severity compared to recurrent parents, with resistance levels approaching or surpassing those of the donor parents. This improvement was particularly evident in introgressed lines derived from *CML539 × DTPYC9-F46* and *CML312 × CLYN261*, confirming the successful transfer and expression of resistance alleles. The high level of resistance observed in introgressed lines of *CML507*,* CZL052*, and *CML574* in heterotic group B further demonstrates the broad effectiveness of the targeted QTLs across diverse genetic backgrounds.

The introgressed lines and their testcrosses not only displayed enhanced MLN resistance but also maintained or improved yield performance under MLN stress. Testcross hybrids derived from introgressed lines showed yield advantages of 2–4 t/ha over their recurrent parents, underscoring the agronomic value of QTL-based conversion. For example, the widely used line *CML312*, known for its strong combining ability, produced BC_4_F_3_ derivatives with markedly reduced MLN severity under artificial inoculation (Fig. 2). Testcrosses of these selections outperformed both donor and recurrent parents for MLN severity and GY under MLN pressure (Figs. 3 and 4). Similarly, *CML539* showed a ~ 50% reduction in MLN severity in its introgressed derivatives, with testcrosses surpassing both parents for yield under MLN stress (Fig. 4) as well as under optimum conditions (Fig. 5). In heterotic group A, the introgressed version of CZL052 line exhibited significant improvement in MLN resistance and nearly two-fold yield gains under severe MLN pressure (Fig. 4). Equally important, the absence of yield penalties or undesirable changes in key agronomic traits under optimum conditions suggests that the introgression process preserved the breeding value of the recurrent parents. GY equivalency under disease-free conditions indicates that the incorporation of MLN resistance QTLs did not negatively impact productivity, husk cover, or resistance to other foliar diseases. This observation is critical, as it addresses a common concern of linkage drag associated with resistance gene introgression^27,45^. The consistent MLN resistance across backgrounds suggests that the introgressed lines carry favorable alleles in three *QTL-hotspot* regions, making them stable and useful for breeding durable MLN resistance (Supplementary Table S1).

Introgression of the MLN-resistance QTLs not only restored resistance but also improved important physiological processes^22^. The resistant segments protected plants from leaf damage and loss of photosynthesis caused by MLN^49^. They may also have improved stay-green, nutrient use, and overall canopy health under disease stress. These improvements helped the plants keep producing energy during grain filling. As a result, the introgressed lines produced higher yields, sometimes even higher than the donor parents. There were no negative changes in major agronomic traits under optimum conditions. This shows that the introgression was precise and kept the plant type unchanged while improving stress tolerance.

Previous studies laid a strong foundation for MLN-resistance breeding^20,23,26,27^. A novel, recessively inherited major-effect QTL (qMLN_06.157) unique to the KS23 background was identified^28^, offering a new and stable source of resistance suitable for developing diagnostic markers. Two major-effect KASP markers on chromosome 3 that consistently predicted MLN resistance across studies were validated and reliably identified resistant introgressed lines under field inoculation, confirming their utility for routine marker-assisted selection^27^.

Building on these advances, the current study focuses on deployment rather than discovery. We introgressed three validated MLN-resistance QTL—including one confirmed by Awata et al.^27^—into fourteen widely used drought-tolerant elite lines in the ESA breeding pipelines. This work uniquely incorporates equivalency testing and comprehensive evaluations of agronomic performance, MLN resistance, and RPG recovery. The introgressed lines exhibited strong MLN resistance, and improved or maintained GY under MLN pressure and optimum conditions. Collectively, these results demonstrate that strategic introgression of multiple MLN-resistance QTL can significantly enhance resistance without compromising agronomic performance.

Although the testcrosses were evaluated in 2017, the MAI-derived lines from this study have remained highly relevant and continue to contribute to ongoing breeding efforts. Subsequent work led to the identification and fine mapping of a major-effect, recessively inherited QTL for MLN resistance from the KS23-6 donor, explaining up to ~ 65% of phenotypic variance^28^. This QTL was pyramided with the dominantly inherited haplotypes reported here, with several BC-derived introgressed lines serving as key parental materials in this process and being integrated into breeding pipelines targeting MLN-prone environments. The resulting lines and hybrids exhibited enhanced and more durable MLN resistance (disease severity < 4 on a 1–9 scale), along with substantial yield gains under MLN pressure. Although formal hybrid release is still underway and subject to national performance evaluation, these MABI-derived inbreds are actively used as donors and parents across multiple breeding programs, contributing to the development and deployment of improved MLN-resistant germplasm.

Future work should prioritize systematic pyramiding of multiple MLN-resistance loci, integrating both major-effect QTL and complementary minor-effect alleles to enhance durability across stress environments^48,49^. The use of high-density markers, haplotype-based selection, and genomic prediction will strengthen multi-locus pyramiding schemes and help identify allele combinations that maximize resistance without compromising agronomic performance. Given the quantitative nature of MLN resistance, further dissection of epistatic interactions among QTL, particularly those on chromosomes 3 and 6, will be essential to understand context-dependent expression and to refine breeding strategies. Integrating genomic, transcriptomic, and metabolomic data with field phenotyping may also clarify the regulatory pathways underlying these interactions. In addition, stacking MLN-resistance QTL with loci conferring drought tolerance, nutrient-use efficiency, and foliar disease resistance represents a promising direction for developing climate-resilient, multi-stress–tolerant hybrids. Such integrative approaches will support the delivery of stable, high-performing germplasm for ESA’s rapidly changing production environments.

## Conclusion

The present study demonstrates the successful introgression of MLN resistance QTLs into elite maize inbred lines from both heterotic groups A and B using MABC. The integration of foreground and background selection ensured efficient recovery of the RPG while retaining favorable alleles for MLN resistance. The results not only confirmed the effectiveness of KASP-based markers in tracking resistance loci but also validated the phenotypic performance of the introgressed lines under artificial MLN inoculation and optimum management conditions. The improved lines developed in this study represent valuable resources for hybrid breeding programs targeting MLN-prone environments. The consistent effectiveness of *qMLN3_133* across genetic backgrounds also suggests its utility as a core target in marker-assisted selection pipelines, whereas the incorporation of additional QTLs from chromosomes 3 and 6 can further broaden resistance. The introgressed lines reported here will serve as strong genetic foundations for breeding programs in sub-Saharan Africa and other MLN-affected regions, ultimately contributing to food security and the sustainability of maize production systems.

## Supplementary Information

Below is the link to the electronic supplementary material.

Supplementary Material 1

Supplementary Material 2

## Data Availability

The data sets generated and analyzed during this study are included in the supplementary materials.
