# Supplementary material for: Introgression of QTL hotspot regions enhances grain yield and maize lethal necrosis resistance in elite maize lines
Source: Sci Rep. 2026 May 21;16:23166. doi: 10.1038/s41598-026-53717-8 (PMC13396450; doi:10.1038/s41598-026-53717-8)
Supplement: Supplementary file 2 — Supplementary Material 2 [file 41598_2026_53717_MOESM2_ESM.docx]

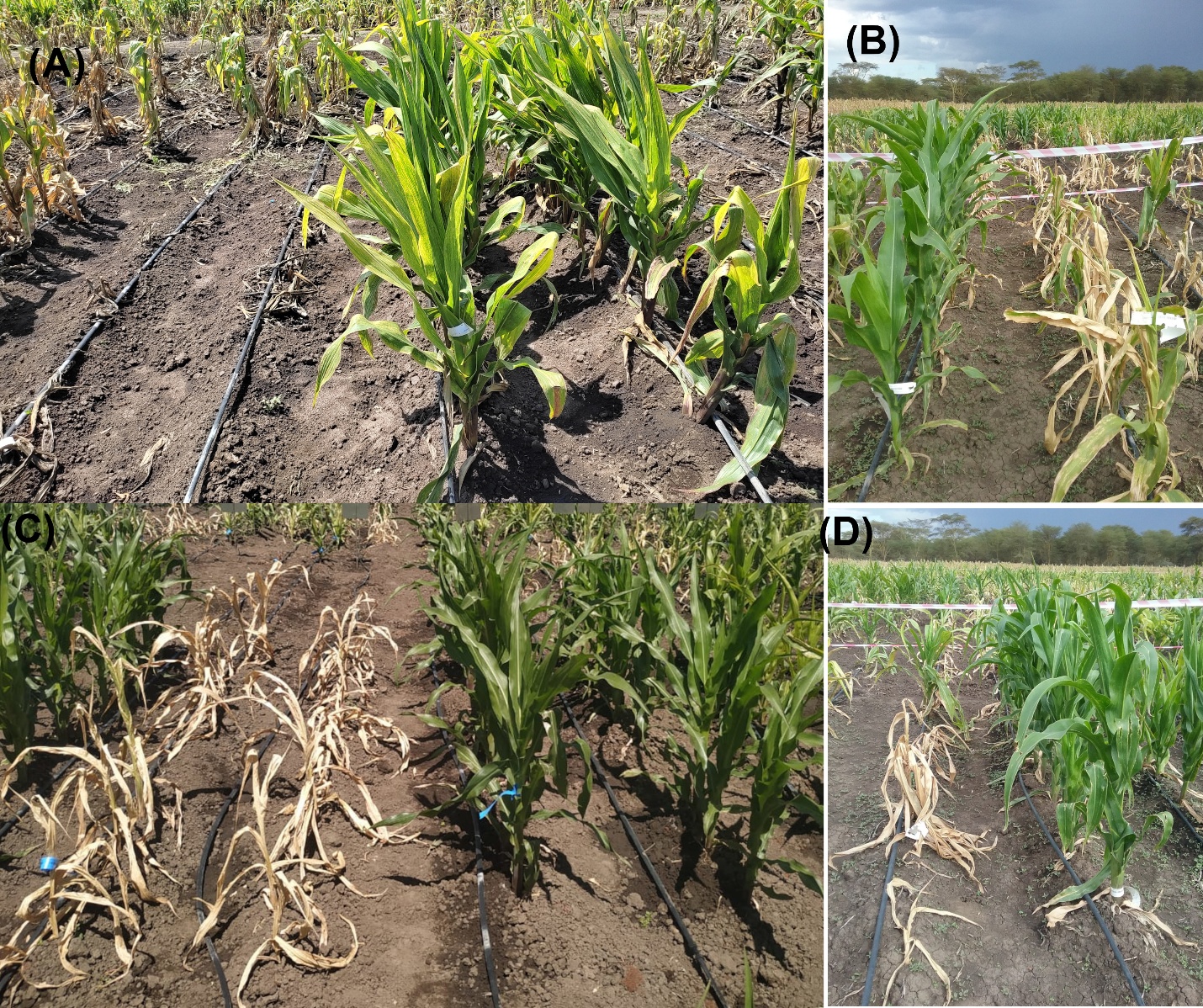


**Figure S1.** Comparative performance of MLN QTL–introgressed maize lines and selected susceptible elite lines under MLN pressure. (A) CML544 (susceptible) versus CML539LNT1 (introgressed); (B) CLRCY034LNT2 (introgressed, resistant) versus CKL5024 (susceptible); (C) CML569 (susceptible) versus DTPWC9-F67LNT2 (introgressed); and (D) CML507 (susceptible) versus CML574LNT1 (introgressed).
